# Simulated Learning for Psychotherapy: A Focus Group Study of Psychiatry Core Trainees in the North West of England

**DOI:** 10.1192/bjo.2024.333

**Published:** 2024-08-01

**Authors:** Sarah Winfield, Anju Sharma

**Affiliations:** 1 Merseycare NHS Foundation Trust, Liverpool, United Kingdom; 2 Manchester NHS Foundation Trust, Manchester, United Kingdom

## Abstract

**Aims:**

Competence in delivering psychotherapy is a mandatory part of the core trainee (CT) curriculum, as mandated by the Royal College of Psychiatrists. CTs who are confident in delivering psychotherapy may provide more meaningful benefit for patients receiving therapy. Simulation is a well-established educational modality but is not widely utilised to teach psychotherapeutic competencies. We aimed to ascertain the views of current CT doctors regarding the use of simulation in psychotherapy training. A greater insight into learning needs would guide development of novel simulated psychotherapy educational resources. This was deemed a priority area for Simulation Based Education in the North West School of Psychiatry.

**Methods:**

Primary data were collected from a virtual focus group of CTs (n = 3) from the North West School of Psychiatry, UK. All CTs in the region were invited to take part, participation was voluntary and informed consent was obtained prior to participation. The focus group was transcribed, analysed and data anonymised to ensure confidentiality.

**Results:**

Participants expressed concerns about embarking on their first psychotherapy case with subthemes relating to: insufficient experience and training in psychotherapy prior to starting a case, the ability to provide an effective intervention for patients and progression through core training. Ideas for how simulated learning may help trainees develop skill in psychotherapy centred around: introductory teaching (with opportunities to watch recordings of simulated patient encounters, examples of psychotherapeutic techniques used as well as using simulation to experience psychotherapeutic supervision) and having opportunities to actively participate in, and observe, individual or group role plays. Engagement with professional actors and psychotherapy faculty during role plays was identified as a priority. Finally, the notion of an introductory Psychotherapy Simulation “one day workshop” was proposed.

**Conclusion:**

There are many ways in which psychiatry CTs' anxieties regarding psychotherapy may be addressed. They may feel better prepared to embark on undertaking therapy clients by engaging in simulated learning opportunities: whether this be actively taking part in role plays and simulations or accessing pre-recorded content of pedagogical simulations outlining underpinning psychotherapeutic theory. The findings from the focus group will be used to inform development of a novel Psychotherapy simulation resource. This will aim to improve the quality of Psychotherapy training in the North West and foster trainees’ confidence in conducting therapy sessions. Psychotherapy faculty will also be interviewed in a subsequent Focus group. Co-production of resources with stakeholders could maximize acceptability and help to maintain ongoing engagement with the project.

